# News

**DOI:** 10.1155/S1463924600000274

**Published:** 2000


Journal of Automated Methods & Management in Chemistry, Vol. 22, No. 5 (September–October 2000) pp. 165–168

### News

### SciQuest.com Announces Opening of UK Office

### Leading global e-marketplace for scientific industry also announces strategic purchasing agreement with Dow and Max-Planck-Institute: opening of Paris and Walldorf, Germany, offices

*Research Triangle Park, NC and Munich, Germany, 10 April 2000 SciQuest.com* (NASDAQ: SQST), a leading business-to-business e-marketplace for products used by pharmaceutical, chemical, biotechnology, industrial and educational organizations, today announced the opening of a local office to serve the UK scientific products marketplace and to initiate the hiring of British senior staff. The announcement was among several issued by SciQuest.com at the Analytica Conference for Analysis, Biotechnology, Diagnostics and Laboratory Technology in Munich, Germany.

Established in 1995, SciQuest.com currently offers researchers and procurement staff the ability to source and purchase approximately one million laboratory products from more than 600 suppliers around the world. In North America, SciQuest.com has used its extensive industry experience to design a marketplace that streamlines the traditionally inefficient scientific products supply chain. The company's marketplace solutions allow buyers of scientific products to cross-search content and purchase products from multiple suppliers with a single order. The company's e-marketplace is distributor-neutral and can be customized and seamlessly integrated with its customers' enterprise systems, including SAP/R3.

SciQuest.com's UK office is to be co-located with the established offices of EMAX Solutions, a SciQuest.com company, at The Nova Building Herschel Street, Slough, Berkshire SL1 1XS. In March, SciQuest.com completed the acquisition of US-based EMAX, for the purpose of creating an integrated e-business solution that manages the entire lifecycle of scientific laboratory products, from selection and procurement through ownership, use and disposal.

'Expanding strategically into key European markets is an important component of the SciQuest.com growth strategy, since most of our established supplier companies and customer research organizations are global,' said Scott Andrews, SciQuest.com co-founder and CEO. 'Our solution streamlines the entire supply chain, allowing scientists to reduce the amount of time spent in supply procurement, while increasing the amount of time available for research and discovery. As the first-mover in the US with this model, we view today's announcement as affirming our commitment to be the leading e-marketplace for the global science industry in the United Kingdom and worldwide.'

### Other News from SciQuest.com

Other news released today in Munich by SciQuest.com includes:

- The launch of pilot purchasing programs at two preeminent European research customers, The Dow Chemical Company and the Max-Planck-Institute. SciQuest.com will expand its strategic North American e-commerce agreement for purchase of scientific products and supplies to Dow sites in Europe. SciQuest.com also will serve as the strategic e-marketplace to conduct pilot testing for designated scientific products purchases for The Max-Planck-Institute of Molecular Cell Biology and Genetics in Germany, integrating with MPI's existing SAP/R3 enterprise systems.
- The appointment of Dr Hans Peter Fatscher as business development manager for Germany. Dr Fatscher holds a PhD from the University of Heidelberg's Institute of Molecular Genetics and brings more than 12 years of sales and management experience for leading European pharmaceutical and biotechnology companies to his new position with SciQuest.com.
- The opening of additional SciQuest.com offices in Paris and Walldorf, Germany. SciQuest.com's French offices will also be co-located with existing EMAX facilities.
- Dr Jason Theodosiou, EMAX vice president and general manager for Europe, is participating this week at the Drug Discovery Technologies 2000 exhibition in Basel, Switzerland (Booth #222, Convention Center, Basel, e-mail: jason.theodosiou @emax.com, telephone: +33 1 46 74 03 83). Previously regional manager for MDL Information Systems' French and Southern European operations, Dr Theodosiou holds a PhD in Chemistry from the University of Marseilles and an MBA from the ESCP in Paris.

Among the 68 research organizations using SciQuest.com as their preferred or exclusive online source for scientific product procurement are: The Dow Chemical Company, DuPont Pharmaceuticals Company, Merck & Co. and The Monsanto Company.

### More about SciQuest.com

SciQuest.com, Inc., is a Web-based, interactive marketplace for scientific and laboratory products used by pharmaceutical, clinical, biotechnology, chemical, industrial and educational organizations worldwide. Sci Quest.com has used its extensive industry experience to design a marketplace that streamlines the traditionally inefficient scientific products supply chain. The company's marketplace solutions allow buyers of scientific products to cross-search and purchase products from multiple suppliers with a single order, and purchase refurbished equipment from SciQuest.com Auctions. In March, SciQuest.com completed the acquisition of Pennsylvania-based EMAX Solution Partners, for the purpose of creating an integrated e-business solution that manages the entire lifecycle of scientific laboratory products, from selection and procurement through ownership, use and disposal. The company's e-marketplace is distributor-neutral and can be customized and seamlessly integrated with its customers' enterprise systems, internal inventory systems and chemical libraries.

*SciQuest.com is headquartered in Research Triangle Park, NC, USA with North American offices in Mountain View, CA, Philadelphia, PA and Plainview, NW. For more information about SciQuest.com please visit www.sciquest.com or call + 1 (919) 659-2100.*

**Kvaerner wins US$70 million contract for environmental modifications to German refinery**

*London, 17 April 2000:* Kvaerner, the Anglo-Norwegian engineering and construction Group, today announced that it has won a lump-sum turnkey, engineering, procurement and construction contract, for modifications to the Holborn Europa Raffinerie in Hamburg, Germany. Under the US$70 million contract, which is due for completion in the summer of 2001, Kvaerner will modify the plant to meet stringent new European Union directives related to the specification of auto fuels.

René Westerduin, Senior Vice President of Kvaerner's Benelux/German operation, states: 'The inherent burden of investment to meet the new fuel specifications means that the refiner must find innovative processing methods, requiring a creative and value-oriented approach. This is where Kvaerner's reputation for innovative engineering solutions provides concrete benefits to the refiner.' The modification, which is one of the first of its kind in Europe will transform diesel fuel components to the required specifications by means of hydro-treatment, and follows the recent completion by Kvaerner of another contract at the refinery.

The refining industry has continually been adjusting to the requirement for change in fuel on the reduction of emissions, not only from the refineries, but also from the fuels themselves. Kvaerner's Benelux/German operation has specific expertise in this field, providing the refiner with a variety of options to conform to the new specifications, and has built up extensive expertise in the safe, high-quality, cost-effective, fast-track execution of these types of projects.

*For further information contact: Paul Emberley, Vice-President, Group Communications, Kvaerner PLC: + 44 (0) 20 7339 1035 or + 44 (0) 468 813090 or www.kvaerner.com.*

**European survey on communications in chemical and life sciences sector launched**

- Industry places priority on community and internal communications.
- Survey indicates cynicism towards sector.

Europe's Chemical and Life Sciences companies could benefit greatly from better communications practices, an international survey has revealed. The Reputation Management European Benchmark (REMB) survey, published today by Edelman Public Relations in Europe, has found that although communications is an important business function for the sector, companies still rank it least important, giving more importance to human resources, research and development, quality control, safety and environment of financial performance.

The Reputation Management European Benchmark survey is the first international study to analyse the impact of corporate communications practices on corporate reputation. It focuses on the ongoing practices of sector communications and how those practices are perceived by key stakeholders.

The survey reveals an element of criticism of and cynicism towards the Chemical and Life Sciences sector across Europe from both the media and other external audiences including various opinion formers who were interviewed as part of the Stakeholders Survey. However, the survey also found that where the companies in the sector communicate more actively they enjoy a better reputation and are more positively perceived by stakeholders. A regression analysis carried out as part of the research demonstrates that companies can and do influence how they are perceived by stakeholders. Communicating more with social organizations representatives, journalists and authorities, is directly related to corporate reputation. Despite this fact the survey found that almost 50% of journalists believe companies do not communicate with them creating an impression of a sector which is 'closed and disrespectful to the environment, to safety or to the community'.

The survey also indicated that a majority of companies in the sector have already developed crisis management devices or measures. Eight-four per cent of companies have crisis management manuals, 71% participate in crisis communications training sessions, while 62% say they have developed crisis communications simulations. In this sector, companies in the UK, France and Spain are those with most experience, except in crisis simulations. Companies in Germany, Italy, Ireland and Belgium appear at the bottom of the range. The sector devotes, on average, 6% of its external relation's budget to crisis management.

One of the striking findings of the report is the strong emphasis which the companies in this sector, right across Europe, place on both internal communications and community relations programmes. These companies see this as their main priority in terms of communications, and community relations is perceived as a key task in the day to day activities of manufacturing plants. The survey pinpoints no national differences when companies are asked if the concerns voiced by a plant's neighbours be justified or not.
In this regard, companies across all seven countries where the survey was conducted understand public concern for the state of the plants. This sensitivity towards local communities is reflected in the priority, which is attached by the sector to community relations.

The REMB survey found that on average 32% of total spend on external relations was dedicated to advertising, a percentage that is even higher in countries such as Germany (40%) and Spain (39%). The second highest budgeted activity after advertising is community relations representing around 15% of average budgets. Internal communications accounted for on average 13% of the total budget, while media relations accounted for 13% of the spend.

There were some differences between countries, e.g. Italy, Germany and the UK place more emphasis on media relations; Ireland places more emphasis on community relations; the UK and Belgium on lobbying; France and Belgium on internal communications; and France and Ireland on research and evaluation.

Over 70% of companies were found to use external public relations services, in most cases on a project by project basis, while 60% produce a written communications plan. These services and practices are especially prevalent in Germany and the UK.

Commenting on the results of the study, John Mahony of Edelman, said

'While companies in the Chemical and Life Sciences sector rate communications as highly as some other business functions they do not attach a high level of importance to it. However its importance to the sector is indicated by the fact that the top communications managers report directly to the head of the company in more than half of the companies and to the marketing manager only in 5% of them. Clearly, where communications is employed internally it operates at the highest level.

By conducting a regression analysis as part of this research we have been able to clearly establish that good communications can clearly improve the perception of companies in these sectors among both media and stakeholders. The Chemical and Life Sciences sectors have experienced difficulties with regard to their image particularly in relation to environmental and safety issues. The findings of the survey reflect those difficulties but also signal the very clear role which better communications practices can play in improving and enhancing the reputation and image of companies in this sector.'

The REMB survey also found that the Chemical and Life Sciences sector clearly recognises the legitimacy and importance of lobbying. Thirty-four per cent of companies have formal institutional/political relations programmes. The Stakeholders survey indicated that the sector can assist in identifying important issues but it was less clear as to the legitimacy of lobbying in the sector.

'The role of the lobbyist is one which has been under review particularly by institutions and the media in many European countries. Clearly, this research highlights again the element of cynicism towards lobbying.' Mahony added.

Commenting on the results of the survey, Mrs Nuria Salo, Director of Masters in Communication to Organisation, Bosch i Gimpera Foundation said; 'Following two years of work in conjunction with the largest European Companies of the Chemical and Life Sciences sectors, universities, NGO's journalists, and Public Administrations from seven countries, the REMB study has confirmed our view that external communications can influence and condition the perception which people have on companies in this sector.'

The survey was conducted among companies in the Chemical and Life Sciences sectors in seven countries (Belgium, Italy, Spain, Ireland, France, Germany and the UK). Key stakeholders in five of these seven countries were also interviewed as part of the survey. These included journalists, authorities with environmental or safety responsibilities, representatives of environmental groups and representatives of scientific institutions. The study has been designed and developed by the University of Barcelona, Bosch i Gimpera Foundation and Edelman PR Worldwide Europe.

*For further information please contact: Simon Scott, Director Media Relations, Edelman Public Relations Worldwide Tel: (0) 2 17344 1253; or Ian O'Doherty, Edelman Public Relations Worldwide Tel: (0) 2 71344 1554*

### Schroder Ventures raises £3 billion European Fund for investment in healthcare and chemicals

*London, 11 May 2000*: Schroder Ventures announces the raising of its second European priviate equity fund, Schroder Ventures European Fund II (SVEF2) which will invest heavily in the healthcare and chemicals buyouts. Just 3 months after its formal launch, Schroder Ventures has received applications from investors for over £ 3 billion, the maximum size set for the fund.

The record speed with which this fund has been rasied reflects the outstanding performance of its predecessor, the Schroder Ventures European Fund (SVEF1). SVEF1, which was raised in 1997, was Europe's first billion dollar private equity fund. It is now fully committed to 21 investments, all companies head-quartered in Europe. Investments include Leica Microsystems (world leading microscopy business), Sirona Dental Systems (world leading dental equipment manufacturer), Mueller & Weygandt (dental consumerables manufacturer), Betts (pharmaceutical packaging business) and Expand Santé (contract sales organization for pharmaceutical sector) – all businesses active in the healthcare sector. SVEF1 has already returned cash to investors equivalent to 134% of the fund within 3 years of the fund's launch.

The investment strategy of the new fund, SVEF2, will follow that of its predecessor, SVEF1. The fund will make both leveraged acquisitions and growth capital investments with a particular focus on the new economy. Seventeen of the first fund's 21 investments have been in the healthcare and technology sectors.

The new fund has been raised from a broad spectrum of investors, 78% of which (by value) are existing investors with Schroder Ventures. Just over half (53%) of the funding came from European investors, with 41% coming from North America and 6% from the Far East.

Since the mid-1990s, Schroder Ventures has adopted a pan-European approach to investment with regional funds and an integrated European team, operating out of four offices in London, Frankfurt, Milan and Paris. The professional team numbers over 50, with 19 partners, who on average have been with Schroder Ventures 9 years.

Schroder Ventures' funds have been investing in European healthcare businesses for 15 years. Notable prior investments include Chiroscience, Shire Pharmaceuticals and Micromass.

Schroder Ventures does not intend to accept any new applications for commitments in the fund.

*For futher information, contact: Schroder Ventures, Charles Sherwood, Partner, London. Tel: + 44 (0) 20 7632 1032; or Amanda Shaw, Marketing Manager, Tel: + 44 (0) 20 7632 1007/44 (0) 7979 706768.*

### Announcement

**New CAMAG catalogue: Planar Chromatography 2000 – modern thin-layer chromatography**

Planar Chromatography is a modern separation technique which is accepted by regulatory agencies and analysts as an extremely flexible, reliable and cost-effective method. It is successfully used in pharmaceutical industry, food analysis, natural products/herbals and many other fields of application.

Besides information on CAMAG products and services the catalogue includes a brief guide to the operating procedure of Planar Chromatography.

The catalogue contents and ancillary information on CAMAG services can also be accessed in the Internet under www.camag.ch

*For further information please contact: Delphine Stoffel, Sales & Marketing Administration, CAMAG, CH-4132 Muttenz/ Switzerland. Tel: + 41 61-467 34 34; Fax: + 41 61-461 07 02; e-mail: info@camag.ch*

